# Evaluation of left ventricular systolic function in different subtypes of pediatric acute lymphoblastic leukemia: a case-control study based on two-dimensional speckle tracking

**DOI:** 10.3389/fphar.2025.1669049

**Published:** 2025-09-22

**Authors:** Yanfeng Yang, Chunyan Fu, Kun Shi, Yonghong Guo, Peng Shi, Hanmin Liu, Ling Gu, Ying Xiong

**Affiliations:** ^1^ West China Second University Hospital, Sichuan University, Chengdu, China; ^2^ Key Laboratory of Birth Defects and Related Diseases of Women and Children (Sichuan University), Ministry of Education, Chengdu, China; ^3^ Chengdu Women’s and Children’s Central Hospital, School of Medicine, University of Electronic Science and Technology of China, Chengdu, Sichuan, China; ^4^ Department of Infectious Diseases and Public Health, City University of Hong Kong, Hong Kong, China; ^5^ Key Laboratory of Chronobiology (Sichuan University), National Health Commission of China, Chengdu, China

**Keywords:** acute lymphoblastic leukemia, echocardiography, two-dimensional speckle tracking imaging, left ventricular systolic function, global longitudinal strain

## Abstract

**Objective:**

To investigate whether 2D-STI can detect the reduction of left ventricular systolic function in children with ALL earlier than conventional echocardiography, and to explore the differences in left ventricular systolic function changes among children with different clinical risk classifications of ALL.

**Methods:**

This study selected 39 (n = 39) children with non-acute lymphoblastic leukemia who were admitted to our hospital from October 2018 to March 2020 (this constitutes the control group), and 39 children with acute lymphoblastic leukemia. Among the children with acute lymphoblastic leukemia, they were divided into the standard-risk group (n = 13), intermediate-risk group (n = 13), and high-risk group (n = 13) according to the CCLG-ALL2008 protocol. Conventional echocardiography was used to measure left ventricular diameter at end-diastole (LVDd), interventricular septal thickness at end-diastole (IVSTd), left ventricular posterior wall thickness at end-diastole (LVPWd), left ventricular ejection fraction (LVEF), peak flow velocity of early (E) and late (A) diastolic filling, and E/A ratio. Two-dimensional speckle tracking imaging was employed to measure longitudinal strain values for statistical analysis.

**Results:**

There were no statistically significant differences in LVDd, IVSTd, LVPWd, LVEF, peak E and A flow velocities, and the E/A ratio among the four groups (*P* > 0.05). The 2D-STI measurement indicators of the control group were not significantly different from those of the standard-risk group (P > 0.05), but showed significant differences compared with the intermediate-risk group and the high-risk group (P < 0.05, P < 0.01); However, among the three groups of children with acute lymphocytic leukemia, except the peak longitudinal strain in the basal segment of the lateral wall, significant differences were observed in the 2D-STI parameters among the groups (*P* < 0.05).

**Conclusion:**

2D-STI is superior to conventional echocardiography for the early detection of reduced cardiac systolic function, and the degree of left ventricular systolic function varies among pediatric ALL patients with different clinical risk classifications.

## 1 Introduction

Acute lymphoblastic leukemia (ALL) is a type of acute leukemia and the most common malignant tumor in children. According to the CCLG-ALL2008 protocol (Sun et al., 2011), the comprehensive diagnosis of ALL takes into account the patient’s age, white blood cell count in peripheral blood, extramedullary leukemia status, cytogenetic characteristics of the tumor cells, and treatment response. Generally, ALL is classified into three risk categories: low risk, intermediate risk, and high risk.

In recent years, with the improvement of cancer diagnosis and treatment, the survival period of cancer patients has been continuously extended and their quality of life has been steadily enhanced (Chen et al., 2016). However, many types of cancer are gradually existing over the long term in a pattern similar to chronic diseases after treatment. At the same time, studies have shown that a significant number of cancer survivors die from non-cancer causes, with cardiovascular diseases being one of the leading causes of death, Including congestive heart failure, cardiomyopathy (Oliva et al., 2021; Xiao et al., 2021), arrhythmia, thrombosis (Tamargo et al., 2015; Chan et al., 2020), etc.

Most current research focuses on cardiac damage secondary to chemotherapy with anthracycline drugs. However, there have been reported cases of acute lymphoblastic leukemia (ALL) where cardiac damage was a primary initial presentation (He and Wang, 1994). Autopsy reports have also revealed that the incidence of leukemic cell infiltration into the heart ranges from 34% to 53% Hu et al., 2009, although symptomatic clinical cases are rare. Leukemia can infiltrate the myocardium, pericardium, and endocardium, with the myocardium being the most commonly affected (Yueshu Chen, 1963; Deng, 1985). This can present as cardiac enlargement, tachycardia, conduction block, pericardial effusion, and heart failure.

Conventional diagnostic methods such as chest X-rays and electrocardiograms (ECGs) have a low detection rate for cardiac abnormalities (Huang et al., 2001). Echocardiography, which is commonly used to evaluate left ventricular systolic function, measures the left ventricular ejection fraction (LVEF). However, studies have shown that an abnormal LVEF value can only be detected when there is significant myocardial damage leading to a noticeable decrease in cardiac function and changes in ventricular morphology and structure, such as myocardial thinning of the ventricular walls or ventricular chamber enlargement (Song and Cheng, 2016). These findings suggest that LVEF may not be sensitive enough to detect early or mild cardiac involvement.

Therefore, finding a method with high sensitivity and specificity for early detection of cardiac damage in children with ALL, and taking proactive measures, is of great importance for reducing cardiac damage.

Two-dimensional speckle tracking imaging (2D-STI) is an advanced echocardiographic technique that tracks the natural acoustic markers—speckles—within the myocardial tissue to obtain detailed information about myocardial motion. The 2D-STI technique analyzes myocardial longitudinal strain, circumferential strain, radial strain, and torsional motion. These parameters can assess the myocardium’s deformation capacity, thereby indirectly reflecting the heart’s functional state. Compared to traditional echocardiography, the advantage of 2D-STI lies in its angle independence, allowing for a more accurate assessment of myocardial function.

Existing studies have shown that 2D-STI can detect cardiac damage in children with ALL earlier than conventional echocardiography. The purpose of this research is to use 2D-STI to evaluate the differences in cardiac damage among ALL patients with different clinical risk classifications. This may provide a more accurate and sensitive method for early intervention, prognosis, pre-chemotherapy cardiac function assessment, early detection of cardiac damage, determination of chemotherapy regimens, and prediction of chemotherapy risks. It also helps in finding new strategies for maintaining and restoring the optimal cardiovascular health of survivors of childhood acute lymphoblastic leukemia.

## 2 Research subjects and methods

### 2.1 Research subjects

A total of 39 children diagnosed with acute lymphoblastic leukemia (ALL) were selected from those admitted to Hospital from October 2018 to March 2020. Inclusion criteria involved classifying all children according to the CCLG-ALL2008 protocol (Sun et al., 2011), dividing them into standard-risk (n = 13), intermediate-risk (n = 13), and high-risk groups (n = 13). Exclusion criteria were: 1) children diagnosed with congenital heart disease, myocarditis, pericarditis, arrhythmias, etc., before treatment; 2) children with systemic autoimmune diseases, diabetes, uremia, and hyperthyroidism causing cardiac dysfunction; 3) factors affecting image quality such as obesity or emphysema.

### 2.2 Research methods

#### 2.2.1 Collection of clinical basic information

The information collected for the study subjects includes age, gender, height, weight, and electrocardiogram (ECG) data.

#### 2.2.2 Echocardiography and 2D-STI measurements

All study subjects underwent conventional echocardiography using a Philips IE-33 echocardiogram machine (S5-1 probe, frequency 1–5 MHz, frame rate ≥50 frames/s). The subjects were instructed to lie on their left side or back (ensuring image clarity; if children under 3 years could not cooperate, oral chloral hydrate sedation was given when necessary) and breathe calmly, with chest lead ECG synchronously recorded.

1. Conventional Echocardiography Measurement Indicators: The same ultrasound technician who was specifically responsible for this study measured the parameters in the absolutely quiet resting state of the children (for children who were young and uncooperative, crying, or emotionally agitated, appropriate sedation was provided), using the 2D biplane Simpson’s method. Each parameter was measured three times, and the average value was taken as the final result. In the parasternal long-axis view, the following measurements were taken: left ventricular diastolic diameter (LVDd), interventricular septum thickness at diastole (IVSTd), left ventricular posterior wall thickness at diastole (LVPWd), and left ventricular ejection fraction (LVEF); The apical four-chamber view was used with pulse Doppler to measure the early diastolic mitral inflow velocity (E peak velocity), the late diastolic mitral inflow velocity (A peak velocity), and to calculate the E/A ratio.

**FIGURE 1:**
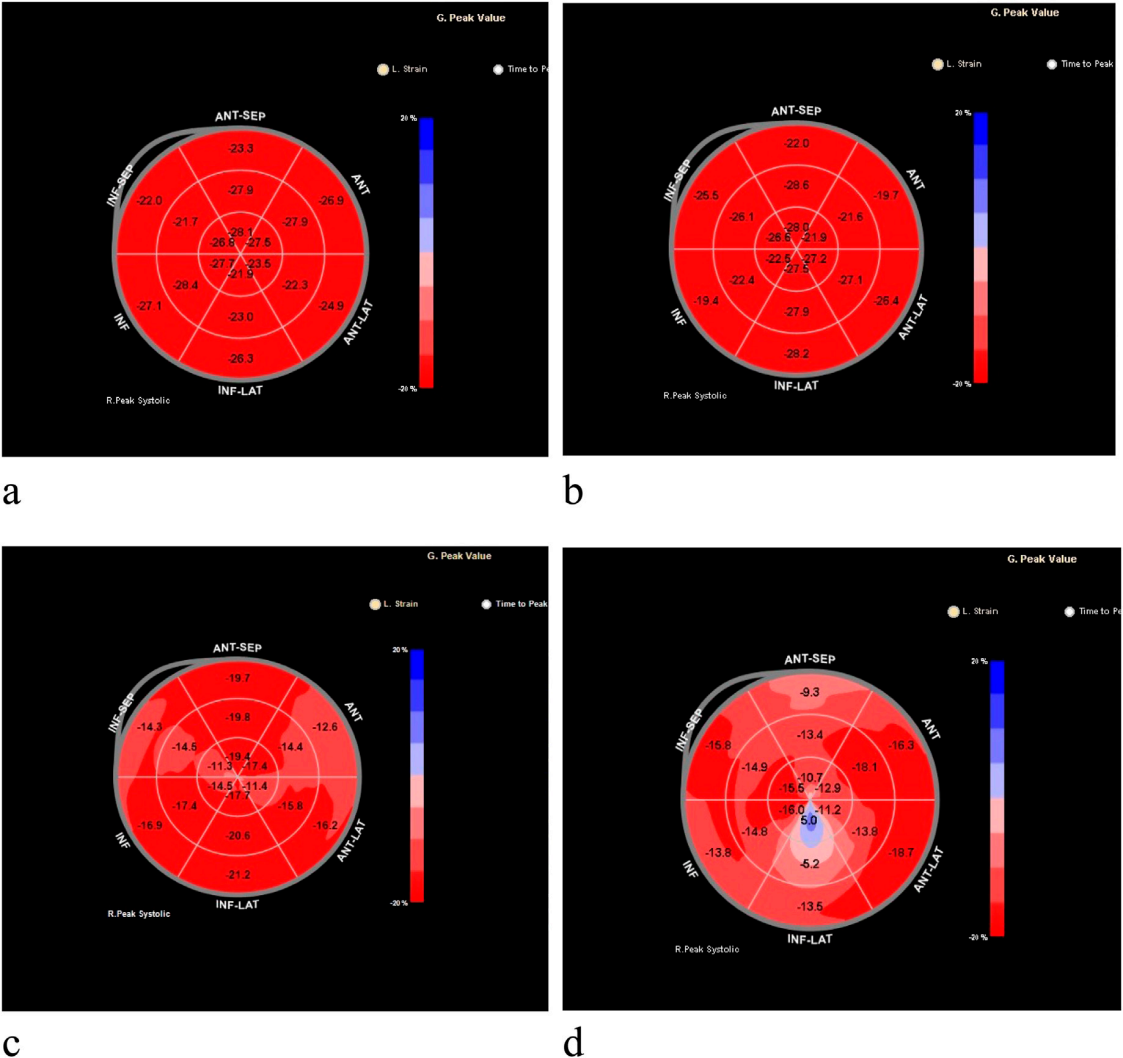
Bull’s-eye plots of the global longitudinal strain of the left ventricle measured by two-dimensional speckle-tracking imaging (2D-STI) technology. **(a)** Represents the left ventricular longitudinal strain for the control group; **(b)** represents the left ventricular longitudinal strain for the standard-risk group; **(c)** for the intermediate-risk group, with a marked decrease in the longitudinal strain in the apical and mid segments of the lateral wall; **(d)** for the high-risk group, with significantly reduced longitudinal strain values across all segments.

2D-STI Measurement Indicators: The ultrasound technician specifically responsible for this study used the same ultrasound equipment and the same measurement method to collect clear views of the left ventricular apex in two-chamber, three-chamber, and four-chamber configurations, as well as the parasternal short-axis view at the level of the mitral valve, papillary muscle, and near the apex, storing three consecutive cardiac cycles, while the children were in a state of absolute quiet rest (for children who were young and uncooperative, crying, or emotionally agitated, appropriate sedation was provided); Using QLAB 9.0 software, the collected two-dimensional images of the heart were analyzed for speckle tracking. At the end of left ventricular diastole, the endocardium of the left ventricle was outlined, adjusting the region of interest to match the myocardial thickness. The software automatically identified and tracked the acoustic speckles within the myocardium, obtaining peak longitudinal strain in the apical four-chamber view (LV AP4 Endo Peak L. Strain, LV AP4 L.S), peak longitudinal strain in the apical three-chamber view (LV AP3 Endo Peak L. Strain, LV AP3 L.S), peak longitudinal strain in the apical two-chamber view (LV AP2 Endo Peak L. Strain, LV AP2 L.S), global longitudinal strain (GLS), and Select the peak longitudinal strain (longitudinal peak strain, LS) of the lateral wall base segment, lateral wall middle segment, lateral wall apical segment, posterior wall base segment, posterior wall middle segment, and posterior wall apical segment. This resulted in an 18-segment bull’s-eye plot of left ventricular longitudinal strain, with colors ranging from deep red to deep blue, corresponding to the GLS from −20% to 20% (Figure 1).

### 2.3 Statistical analysis

All data were analyzed using SPSS statistical software, version 24.0. For quantitative data that followed a normal distribution, means ± standard deviations were used to express the results. Comparisons among the three groups were performed using post-hoc tests following one-way ANOVA. For quantitative data not following a normal distribution, the median (interquartile range) was used for expression, and the Mann-Whitney U test was applied in cases of heterogeneity of variance.

Categorical data were expressed as percentages, and comparisons between groups were made using Fisher’s exact test. A p-value of less than 0.05 was considered statistically significant.

## 3 Results

### 3.1 General information

During the study period, a total of 39 children with Acute Lymphoblastic Leukemia (ALL) meeting the inclusion criteria were collected. All children were clinically classified according to the CCLG-ALL2008 protocol (Yueshu Chen, 1963), dividing them into standard-risk (n = 13), intermediate-risk (n = 13), and high-risk (n = 13) groups. There was no statistically significant difference in age, gender, or body weight among the three groups (*P* > 0.05), Table 1.

**TABLE 1:** Basic information of children with control, all in standard risk, intermediate risk, and high risk groups.

Iterm	Control	Standard risk	Intermediate risk	High risk	Statistic	P
Age (years)	4.34 ± 3.04	4.62 ± 3.64	3.62 ± 1.76	4.96 ± 3.98	F = 0.427	0.735
Gender (male/female)	20/19	8/5	6/7	9/4	—	0.756^a^
Weight (kg)	18.03 ± 7.24	19.50 ± 14.07	15.58 ± 4.64	20.15 ± 12.50	F = 0.615	0.607
Electrocardiogram (abnormal/normal)	0/39	0/13	1/12	0/13	—	0.5^a^

^a^ Fisher’s exact test used for statistical analysis.

### 3.2 Comparison of conventional echocardiographic measurement indicators among groups

**FIGURE 2:**
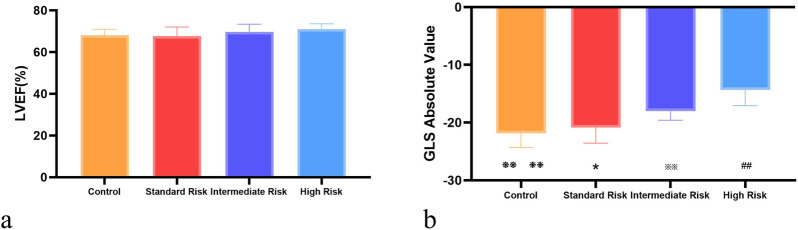
Intergroup comparison of Left Ventricular Ejection Fraction (LEVF %) and Global Longitudinal Strain (GLS). **(a)** shows the comparison of LEVF (%) across control, standard-risk, intermediate-risk, and high-risk groups; **(b)** displays the comparison of GLS across the same risk groups. Control vs. Standard Risk, ❃P < 0.05, ❃❃ P < 0.01; Control vs. Intermediate Risk, ❉P < 0.05, ❉❉ P < 0.01 P < 0.01; Control vs. High Risk: ❈P < 0.05, ❈❈ P < 0.01; Standard Risk vs. Intermediate Risk, *:P < 0.05, **:P < 0.01; Standard Risk vs. High Risk, #:P < 0.05, ##: P < 0.01; Intermediate Risk vs. High Risk, ※:P < 0.05, ※※: P < 0.01.

There was no statistically significant difference among the standard-risk, intermediate-risk, and high-risk groups in terms of LVDd, IVSTd, LVPWd, LVEF, E peak velocity, A peak velocity, and E/A ratio (*P* > 0.05). Table 2 and Figure 2a.

**TABLE 2:** Comparison of conventional echocardiographic measurement indicators among all children in control, standard risk, intermediate risk, and high risk groups.

Iterm	Control	Standard risk	Intermediate risk	High risk	Statistic	P
LVEF (%)	68.11 ± 2.79	67.64 ± 4.41	69.67 ± 3.67	69.87 ± 2.31	1.87	0.142
LVDd (mm)	34.15 ± 6.63	33.19 ± 6.63	32.4 ± 3.24	33.50 ± 5.07	0.547	0.652
IVS(mm)	4.36 ± 0.97	3.97 ± 1.172	3.72 ± 0.66	4.28 ± 1.37	1.454	0.234
LVPW(mm)	4.43 ± 1.16	3.93 ± 1.13	3.72 ± 0.66	4.28 ± 1.37	1.578	0.202
E (m/s)	1.08 ± 0.11	1.04 ± 0.12	1.06 ± 0.18	1.06 ± 0.20	0.257	0.856
A (m/s)	0.68 ± 0.06	0.69 ± 0.16	0.75 ± 0.18	0.72 ± 0.15	1.29	0.284
E/A	1.60 ± 0.13	1.58 ± 0.33	1.52 ± 0.47	1.51 ± 0.23	0.587	0.0.625

### 3.3 Comparison of 2D-STI measurement indicators among groups

There were no significant differences in the 2D-STI measurement indicators of the control group compared with the intermediate-risk group (P > 0.05). In the peak longitudinal strain within the apical four-chamber view, peak longitudinal strain within the three-chamber view, peak longitudinal strain within the two-chamber view, and overall longitudinal strain of the left ventricle, the middle-risk group and the high-risk group showed significantly lower values compared with the control group, and the differences were statistically significant (P < 0.05, P < 0.01), as shown in Table 3. The peak longitudinal strain values in the lateral apical segment, middle posterior segment, and apical posterior segment of the side wall, and the middle posterior segment and basal posterior segment of the posterior wall were significantly lower in the middle-risk group and the high-risk group compared with the control group, and the differences were statistically significant (P < 0.05, P < 0.01), as shown in Table 4.

**TABLE 3:** Comparison of global left ventricular longitudinal strain and longitudinal strain at the apical two-chamber, three-chamber, and four-chamber views among different groups.

Group	LV AP2 L.S (%)	LV AP3 L.S (%)	LV AP4 L.S (%)	GLS
Control Standard Risk	−21.94 ± 2.42 −20.8 ± 2.75	−21.36 ± 2.88 −20.12 ± 3.51	−21.15 ± 2.62 −21.78 ± 3.66	−21.07 ± 2.14 −20.85 ± 2.70
Intermediate Risk	−18.12 ± 2.42^※※❉❉^	−17.65 ± 2.54^※※❉❉^	−18.23 ± 2.06^*※❉^	−18.00 ± 1.58^*※※❉❉^
High Risk	−14.58 ± 3.33^##❈❈^	−13.4 ± 3.47^##❈❈^	−15.07 ± 2.92^##❈❈^	−14.37 ± 2.69^##❈❈^

Control vs. Standard Risk, ❃*P* < 0.05, ❃❃ *P* < 0.01; Control vs. Intermediate Risk, ❉*P* < 0.05, ❉❉ *P* < 0.01; Control vs. High Risk: ❈*P* < 0.05, ❈❈ *P* < 0.01; Standard Risk vs. Intermediate Risk, *:P < 0.05, **:P < 0.01; Standard Risk vs. High Risk, #:P < 0.05, ##: P < 0.01; Intermediate Risk vs. High Risk, ※:P < 0.05, ※※: P < 0.01.

**TABLE 4:** Comparison of peak longitudinal strain of the left ventricular lateral wall and posterior wall at apical, mid, and basal segments.

Group	LV AP2 L.S (%)	LV AP3 L.S (%)	LV AP4 L.S (%)	GLS
Control Standard risk	−21.94 ± 2.42 −20.8 ± 2.75	−21.36 ± 2.88 −20.12 ± 3.51	−21.15 ± 2.62 −21.78 ± 3.66	−21.07 ± 2.14 −20.85 ± 2.70
Intermediate risk	−18.12 ± 2.42^※※❉❉^	−17.65 ± 2.54^※※❉❉^	−18.23 ± 2.06^*※❉^	−18.00 ± 1.58^*※※❉❉^
High risk	−14.58 ± 3.33^##❈❈^	−13.4 ± 3.47^##❈❈^	−15.07 ± 2.92^##❈❈^	−14.37 ± 2.69^##❈❈^

Control vs. Standard Risk, ❃*P* < 0.05, ❃❃ *P* < 0.01; Control vs. Intermediate Risk, ❉*P* < 0.05, ❉❉ *P* < 0.01; Control vs. High Risk: ❈*P* < 0.05, ❈❈ *P* < 0.01; Standard Risk vs. Intermediate Risk, *:P < 0.05, **:P < 0.01; Standard Risk vs. High Risk, #:P < 0.05, ##: P < 0.01; Intermediate Risk vs. High Risk, ※:P < 0.05, ※※: P < 0.ss01.

And in the three groups of acute lymphoblastic leukemia, Except for the peak longitudinal strain of the lateral basal segment, the differences in 2D-STI measurement indicators among the groups were statistically significant (*P* < 0.05). The high-risk group had significantly lower values of all longitudinal strain measurements compared to the standard-risk group (*P* < 0.01), and the reduction was most pronounced, as seen in Tables 3, 4; Figure 1. When compared to the intermediate-risk group, the high-risk group showed a significant decrease in peak longitudinal strain in the apical four-chamber view, peak longitudinal strain in the three-chamber view, peak longitudinal strain in the two-chamber view, global longitudinal strain, and mid-segment strain of the posterior wall, and basal and mid-segment strain of the lateral wall (*P* < 0.05). The comparison between the standard-risk and intermediate-risk groups revealed significant differences only in peak longitudinal strain in the apical four-chamber view, global longitudinal strain, and apical and mid-segment strain of the lateral wall (*P* < 0.05), as shown in Tables 3, 4; Figure 1. Overall, in acute lymphoblastic leukemia, a progressive decrease in global longitudinal strain across the three groups was observed, with the differences being statistically significant (*P* < 0.05),as depicted in Figure 2b.

## 4 Discussion

Two-dimensional speckle tracking imaging (2D-STI) utilizes the principle of speckle tracking to automatically identify speckle structures on two-dimensional images. This technique facilitates the acquisition of myocardial displacement and deformation by tracking the movement of these speckles. It enables frame-by-frame tracking of speckle movement in conjunction with the myocardium’s motion from any angle and the relative movement between speckles, thus allowing for the rapid quantification of longitudinal strain values in various myocardial segments. Compared to traditional echocardiography, STI exhibits a lower dependency on cardiac load, chamber size, and heart geometry, which enhances its accuracy in assessing myocardial contractility (Zhu et al., 2000). It is not limited to measuring localized myocardial strain; it can also analyze the overall strain Roberts et al., 1968. The study of Urheim S et al. (Urheim et al., 2000; Cheung et al., 2010; Mori et al., 2007) also confirmed that 2D-STI can detect abnormal deformation caused by minor myocardial injury even if the conventional ultrasound indicators are normal. Moreover, this technology has good repeatability, is user-friendly, and produces precise results. It has the potential for early detection of subclinical cardiac dysfunction, making it an excellent quantitative technique for cardiac function assessment.

Changes in cardiac function in patients with leukemia are difficult to detect in the early stage, and LVEF alone is not enough to evaluate. Previous studies (Poterucha et al., 2012; Panoulas et al., 2014) have shown that in the early stage of cardiac function impairment, most patients are in a subclinical state, and no definite positive findings have been found in ECG, myocardial enzyme spectrum, left ventricular ejection fraction (LVEF) and other indicators. 2D-STI can detect minor lesions in local and global myocardial function at an early stage, as well as cardiac systolic function impairment.

For pediatric leukemia patients, the abnormal proliferation and accumulation of leukemia cells in the bone marrow suppress normal hematopoiesis, leading to anemia, thrombocytopenia, and neutropenia. Consequently, leukemia patients often suffer from varying degrees of anemia. Severe anemia can cause hypoxia in myocardial cells, leading to fatty degeneration and the development of anemic cardiomyopathy, which further exacerbates myocardial damage. In turn, myocardial damage can worsen anemia. This interplay can lead to a progressive decline in cardiac function (Ma et al., 2014). Furthermore, infiltration of leukemia cells into myocardial tissues can cause hemorrhage and edema in the heart muscles, while metabolic disorders and microcirculation changes in leukemia patients further reduce heart function.

To date, there is no worldwide consensus on the clinical risk criteria for pediatric acute lymphoblastic leukemia (ALL) patients. In principle, the criteria should integrate the patient’s age at diagnosis, peripheral blood white cell count, extramedullary leukemia status, genetic characteristics of the tumor cells, and treatment response. Therefore, the extent of leukemia cell infiltration into myocardial cells and the degree of anemia may be the reasons for the differences in the impact of various clinical risk stratifications on left ventricular systolic function in ALL. Houx-iang Solomon et al., 1994 conducted a study on rats with acute myocardial infarction, which indicated that anemia could increase the area of myocardial infarction. Transfusion to achieve a hemoglobin level of 100 g/L was shown to reduce the infarct size. While 2D-STI can distinguish systolic strain between normal and ischemic myocardium, and can also assess infarct size to add important diagnostic and prognostic information (Leitma et al., 2004; Joli et al., 2009).

In this study, by using conventional echocardiography to measure left ventricular end-diastolic diameter (LVDd), interventricular septal thickness at end-diastole (IVSTd), left ventricular posterior wall thickness at end-diastole (LVPWd), left ventricular ejection fraction (LVEF), peak velocity of the E wave, peak velocity of the A wave, E/A ratio, and by using 2D speckle tracking imaging (2D-STI) to measure longitudinal strain values in each group, it was confirmed that 2D-STI can detect subtle myocardial lesions in both localized and overall function earlier than conventional echocardiography, identifying early reductions in cardiac contractility. Additionally, the study found that children with different clinical risk stratifications of acute lymphoblastic leukemia (ALL) had varying degrees of left ventricular systolic function change, with statistically significant differences. The differences were most pronounced between the standard risk group and the high-risk group, and less so with the intermediate-risk group. The global longitudinal strain (GLS) showed a stepwise decreasing trend from standard risk to intermediate to high risk, with statistically significant differences. However, there were no statistically significant differences in the peak longitudinal strain values of the lateral basal segments between the groups, which may be due to the coordination of overall cardiac motion where specific myocardial segments play a primary or secondary role at certain phases of the cardiac cycle (Ren et al., 2010). It may also be because the rate of myocardial deformation is affected by the movement of the whole myocardium and adjacent segments, which cannot truly reflect myocardial function, and is related to local myocardial stress and structural differences (Marwick, 2006; Wei et al., 2014).

Furthermore, the study found that the LVEF of all children with ALL was within the normal range, suggesting that overall heart function was normal. This could be because when a certain myocardial segment experiences contractile dysfunction, the surrounding normal myocardium, due to passive stretching, makes it appear similar to normal myocardium in overall cardiac motion. Therefore, the change in the left ventricular overall systolic function parameter LVEF is not significant.

However, this study still has some limitations. Due to the special timing of the project’s implementation, the hospital’s patient flow was low, and combined with the young age of the patients and their low cooperation rate, it led to difficulties in sample collection and some cases being lost. As a result, the overall sample size was small. However, we can increase our sample size in the subsequent follow-up studies to enhance the value of the research. Moreover, in the subsequent research, we will provide the changes in 2D-STI measurement indicators after the use of chemotherapy drugs, as well as the correlation analysis between the cumulative amount of chemotherapy drugs and the 2D-STI measurement indicators, in order to help us select safer and more effective chemotherapy drug doses, provide a safer treatment plan for children with acute lymphoblastic leukemia, and further reduce the damage of chemotherapy drugs to the heart, prolong survival time, and improve survival quality.

In conclusion, For cardiac damage in leukemia patients, appropriate treatment should be given according to the varying degrees of damage. Consequently, the identification of a method with high sensitivity and specificity for the early detection of cardiac damage in children with ALL and for understanding the extent of this damage is crucial. Proactive measures must be taken to minimize cardiac injury, as well as to evaluate cardiac function prior to chemotherapy, to determine the appropriate chemotherapy regimen, and to predict the risks associated with chemotherapy. Providing a more accurate, sensitive, and targeted approach is of great significance.

## Data Availability

The original contributions presented in the study are included in the article/supplementary material, further inquiries can be directed to the corresponding author.
